# Occurrence of oxytetracycline residues in milk samples from Palakkad, Kerala, India

**DOI:** 10.14202/vetworld.2020.1056-1064

**Published:** 2020-06-11

**Authors:** M. Asif Hebbal, C. Latha, K. Vrinda Menon, Jolly Deepa

**Affiliations:** 1 Department of Veterinary Public Health, College of Veterinary and Animal Sciences, Mannuthy, Thrissur, Kerala, India; 2 Department of Veterinary Public Health, Kerala Veterinary and Animal Sciences University, Pookode, Wayanad, Kerala, India

**Keywords:** enzyme-linked immunosorbent assay, high-performance liquid chromatography, microbial inhibition assay, oxytetracycline residues, Palakkad, pooled milk

## Abstract

**Background and Aim:**

Food of animal origin such as milk is vital for nutritional security and should be free of any antibiotic residues due to its public health significance. We designed a study aiming to determine the occurrence of antibiotic residues and in further levels of oxytetracycline residues in pooled raw milk samples from Palakkad district, Kerala.

**Materials and Methods:**

We collected pooled raw milk samples were collected from Alathur, Chittoor, and Palakkad blocks of Palakkad district, Kerala. A total of 215 samples were screened for antibiotic residues by microbial inhibition assay (MIA) and the positive samples were subjected to enzyme-linked immunosorbent assay (ELISA) to determine oxytetracycline residues, this was further confirmed using high-performance liquid chromatography (HPLC).

**Results:**

We found that out of the 215 pooled raw milk samples screened for antibiotic residues using MIA, 22 samples (10.23%) were positive for antibiotic residues from Palakkad, Kerala. Out of these 22 samples, five (2.33%) were positive for oxytetracycline residues. We further calculated the mean concentration of oxytetracycline residues in these five samples and estimated it to be 201.00±41.25 ng/mL and 272.11±53.21 ng/mL using ELISA and HPLC, respectively. On analyzing these five samples, we found that four samples (1.86%) exceeded the maximum residue limits level of 100 ng/mL for oxytetracycline residues in milk as specified by Codex Alimentarius Commission/Food Safety and Standards Authority of India (FSSAI).

**Conclusion:**

This study revealed that the occurrence of oxytetracycline residues in pooled raw milk samples in the Palakkad district of Kerala. Hence, there is a need for surveillance and monitoring of antibiotic residues in milk due to its impact on public health to ensure consumer safety.

## Introduction

Milk is an indispensable part of our diet, ­consumed by people of all ages because of its high bioavailability and contribution to the daily recommended intake of nutrients [1]. Dairy livestock rearing offers food, nutritional security, socio-economic development, and women empowerment in India. With the rise in demand for foods of animal origin and growing export potential, the livestock dairy industry is rapidly intensifying along with an increase in the administration of antibiotics in food-producing animals to prevent and treat diseases [2,3]. Although the use of antibiotics for growth promotion is banned since 2006 by the European Union (EU), India has been one of the top consumers of agricultural antibiotics worldwide, accounting for 3% of the global consumption and this use is estimated to double by 2030 [4]. The global estimate of antimicrobial agents used in animals in 2014 adjusted for animal biomass was 98.97 mg/kg [5].

Extensive use of antimicrobials in food-producing animals has triggered the development of antimicrobial resistance (AMR) in these animals and the environment, which presently is an international concern. Livestock-associated Methicillin-resistant *Staphylococcus aureus*, foodborne pathogens such as *Salmonella* spp., *Campylobacter* spp., and *Escherichia coli* can be transmitted from these animals to humans through food and other transmission routes [6-9]. The consumption of milk with antibiotic residues may pose a potential health risk for consumers. These residues can cause toxic effects such as anaphylactic reactions, carcinogenicity (oxytetracycline, sulfamethazine, and furazolidone), nephropathy (gentamicin), mutagenicity, reproductive disorders, bone marrow toxicity (chloramphenicol), hepatotoxicity, and immunopathological effects in humans [10,11]. The presence of residues in milk may further lead to failure of starter culture, disrupting manufacture of yogurt, cheese, and other dairy products which can cause significant ­economic losses for the dairy industry [12].

Oxytetracycline is a widely used antimicrobial agent of the tetracyclines group in animal health for therapeutic and prophylactic purposes [13,14]. The indiscriminate and non-prudent usage of oxytetracycline and other antibiotics by dairy farmers in India could lead to the occurrence of antibiotic residues in milk. The prevalence of undernourishment of people in India is estimated to be 195 million in 2017 [15]. Thus, milk is essential to meet the daily nutritional requirement and it must be of good quality and safe for human consumption.

In this study, we aimed to determine the occurrence of antimicrobial residues and especially the level of oxytetracycline residues in pooled raw milk samples of Palakkad district, Kerala.

## Materials and Methods

### Ethical approval and Informed consent

Ethical approval was not necessary for this study. Informed consents of the dairy farmers and man­agement of milk cooperative societies in the study area were obtained.

### Study location

The samples were obtained from the cooperative societies in Alathur, Chittoor, and Palakkad blocks of Palakkad district, Kerala. Palakkad district belongs to the Malabar region of Kerala and it is subdivided into 13 Block Panchayats. The milk production of Kerala was 25.76 million tonnes with the per capita availability 192 g/day in the year of 2017-2018.

### Sample collection

The study was conducted from November 2019 to March 2020. A total of 215 pooled raw milk samples (150 mL) comprising 75, 70, and 70 samples from Alathur, Chittoor, and Palakkad blocks, respectively, were collected in sterile sample containers under aseptic conditions. The samples were brought to the laboratory under the refrigerated condition and stored in a deep freezer at −20°C until analysis.

### Bacterial culture and media

The standard cultures of *Bacillus cereus* MTCC 430, *Bacillus subtilis* MTCC 441, *E. coli* MTCC 3221, and *Geobacillus stearothermophilus* MTCC 38 were obtained from Microbial Type Culture Collection and Gene Bank (Chandigarh, India) and were used as a reference culture. The culture media used were antimicrobial inhibitor test agar at pH 6 (Bc6), pH 7.2 (Bs7.2), and pH 8 (Ec8) and the Diagnostic Sensitivity Test (DST) agar (HiMedia, Mumbai, India).

### Screening of antibiotic residues in milk

The pooled raw milk samples were screened for antibiotic residues using microbial inhibition assay (MIA) based on the work by Gaudin *et al*. [16]. The MIA was designed with four test organisms *B. cereus, B. subtilis, E. coli*, and *G. stearotherm philus* in four different media Bc6, Bs7.2, Ec8, and DST, respectively. The tested microorganisms were cultured in nutrient broth (HiMedia) at 37°C for 24 h. The bacterial suspension turbidity was adjusted to 0.5 McFarland standard and inoculated onto test media using sterile swabs using standard procedure. The milk samples were heated at 80°C for 5 min before analysis. The sterile disks (HiMedia) of diameter 6 mm were dipped in the milk samples and placed in all four-test media using sterile forceps. Then, the plates were incubated for 18-24 h. After incubation, plates with a zone of inhibition of 12 mm in at least one plate were considered as positive for antibiotic residues.

### Determination of oxytetracycline residues in milk using enzyme-linked immunosorbent assay (ELISA)

The pooled raw milk samples positive for antibiotic residues in MIA were subjected to the MaxSignal^®^ oxytetracycline ELISA test kit (BIOO Scientific Corp., Texas, USA) to determine the oxytetracycline residues. The test kits contained 12 strips with eight removable wells each, standards of different concentrations (negative control, 0.15, 0.375, 0.75, 1.5, and 4.5 ng/mL), anti-tetracycline antibody, horseradish peroxidase (HRP) conjugated secondary antibody, wash buffer, 3,3’,5,5’-tetramethylbenzidine (TMB) substrate, and stop buffer.

The milk samples (1 mL) were prepared by the addition of sample extraction buffer (3 mL) and centrifuged at 4000 g for 10 min. The supernatant (200 μL) was mixed with 25 μL sample balance buffer and 275 μL sample diluent and the mixture was vortexed for 1 min. A volume of 75 µL of each standard solution or prepared sample was added to each well in duplicates followed by 100 µL of anti-tetracycline antibody solution. The plate was mixed well by gently rocking for a minute manually and incubated at room temperature for 50 min. Each well of the ELISA plate was washed using ImmunoWash™ 1575 microplate washer (Bio-Rad Laboratories, California, USA) with 250 µL of wash buffer and was repeated 3 times. Thereafter, 150 µL of peroxidase enzyme-conjugated secondary antibody was added to each well and the plate was washed after incubation for 20 min. Further, 100 µL of TMB substrate was added to each well and the plate was incubated in the dark for 15 min at room temperature. Finally, 100 µL of stop buffer was added to stop the reaction and absorbance was recorded at 450 nm primary filter and 630 nm differential filter wavelengths immediately with a microplate reader (Bio-Rad Laboratories, California, USA).

The calibration standard curve was plotted between concentrations and relative absorbance of the standard solutions on a logarithmic curve. To obtain the actual concentration of antibiotic residues in the milk samples, the concentration obtained from the calibration curve was further multiplied by the dilution factor. The results were calculated by obtaining the optical density values and calculating the relative absorbance (%).


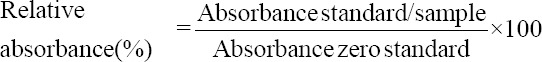


### Confirmation of oxytetracycline residues in milk using high-performance liquid chromatography (HPLC)

The samples positive for oxytetracycline ­residues in milk using ELISA were confirmed using Ultra-HPLC-Diode Array Detector (UHPLC-DAD). The UltiMate™ 3000 Rapid Separation UHPLC system (Thermo Fisher Scientific, USA) equipped with a DAD-3000RS (deuterium and tungsten light source) and a Chromeleon™ CDS version 6 software for data analysis were used. The Acclaim™ C18 column with 4.6×250 mm size, 120 Å pore size, and 5 µm internal diameters was used for the separation of analytes.

### Standard and reagents

The analytical grade standard of oxytetracycline (99%) was obtained from HiMedia, India, and was stored at −20°C under dry conditions which were brought at room temperature before preparing stock and working solutions. Analytical grade oxalic acid and trichloroacetic acid; HPLC grade acetonitrile and methanol were purchased from Merck, Germany. HPLC grade water from Milli-Q system from Merck Millipore, USA, was used for the preparation of the mobile phase.

### Sample preparation

The milk sample (1 mL) was mixed with freshly prepared 10% (w/v) trichloroacetic acid solution (1 mL) and the mixture was vortexed for 30 s. The tube was centrifuged at 12,000 g for 10 min and the supernatant was filtered through a 0.22 µm disposable syringe filter. A 20 µL volume of the filtrate was directly injected into the HPLC system for analysis.

### HPLC analysis

The HPLC method for the detection and quantitation of oxytetracycline residues was based on the method described by Cinquina *et al*. [17] with slight modifications. The mobile phase for oxytetracycline detection comprised 0.03 *M* oxalic acid, acetonitrile, and methanol in the ratio of 70:15:15. Isocratic elution with C18 column was carried out and the retention time was 5.4 min for oxytetracycline. The flow rate was fixed at 1 mL/min with a column temperature set at 40°C. The detection wavelength was set at 354 nm in DAD for maximum sensitivity.

The oxytetracycline standard stock solution (100 mg/mL) was prepared by dissolving 1 mg of the oxytetracycline standard in 10 mL of the mobile phase. Working standard solutions were prepared by diluting the stock solution with the mobile phase to obtain concentrations of 50, 100, 250, 500, 1000, and 2000 ng/mL. A standard calibration curve was prepared by plotting average of the peak area against standard concentrations. The linearity of the calibration curve was evaluated using least square linear regression analysis and the coefficient of determination (R^2^) was determined.

### Recovery study

The validation of the HPLC method for the detection and quantitation of oxytetracycline residues in milk was conducted by recovery study. The blank milk samples spiked at three fortification levels 200, 1000, and 2000 ng/mL were subjected to extraction and analysis. The precision and accuracy of the method were measured as % relative standard deviation (% RSD) and % recovery, respectively. The intra-assay and inter-assay precision was evaluated with the sample extraction method operating under the same conditions over 1 day and 3 consecutive days, respectively. The limit of detection (LOD) and the limit of quantitation (LOQ) of the method were also calculated.

### Statistical analysis

The data obtained were subjected to statistical analysis using SPSS version 24.0, (IBM, New York, USA). The significance of the occurrence of antibiotic residues among different blocks was analyzed using the Chi-square test for multiple proportions. Statistical analysis of quantitative data (ELISA and HPLC) was performed by estimating mean as a measure of central tendency and range as a measure of dispersion. The values of ranges and means for residues of various antibiotics in test samples were calculated.

## Results

**Figure-1:**
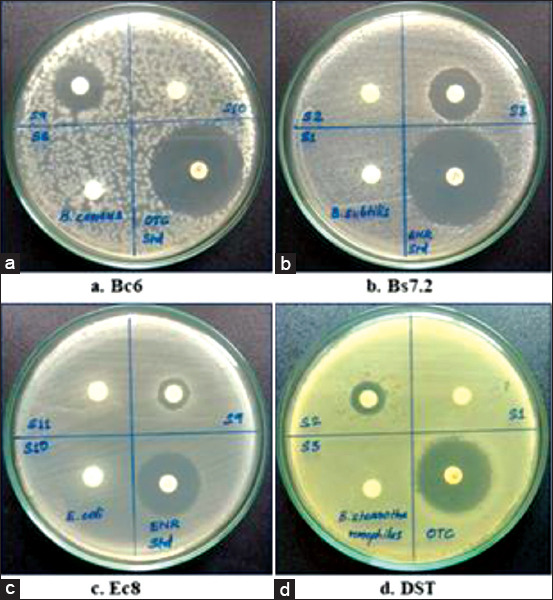
Milk samples positive for antibiotic residues using microbial inhibition assay (a) Bc6-*Bacillus cereus*, (b) Bs7.2-*Bacillus subtilis*, (c) Ec8-*Escherichia coli*, (d) DST-*Geobacillus stearothermophilus*.

Out of the 215 pooled raw milk samples screened for antibiotic residues using MIA, 22 samples (10.23%) were found positive for antibiotic residues from Palakkad, Kerala. The positive pooled raw milk samples which resulted in the production of the inhibitory zone above 12 mm in each of the tested microorganisms are shown in Figure-1. The occurrence of antibiotic residues in pooled raw milk samples from Alathur, Chittoor, and Palakkad was 12%, 11.43%, and 7.14%, respectively. The statistical analysis using the Chi-square test for multiple proportions revealed no significant difference in the occurrence of antibiotic residues in milk samples between blocks (Table-1).

**Table-1:** Occurrence of antibiotic residues in pooled raw milk samples using MIA.

Sl. no.	Blocks	Samples analyzed	Positive samples

			Number (%)
1.	Alathur	75	9 (12)
2.	Chittoor	70	8 (11.43)
3.	Palakkad	70	5 (7.14)
4.	Total	215	22 (10.23)
χ^2^ value		1.09	
p-value		0.58	

MIA=Microbial inhibition assay

**Figure-2:**
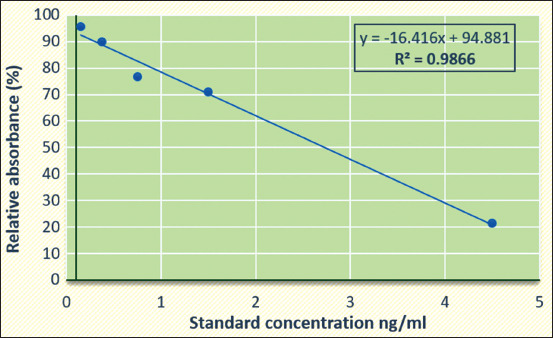
Standard calibration curve for oxytetracycline by enzyme-linked immunosorbent assay.

Standardization of ELISA for the detection and quantitation of oxytetracycline residues in milk was performed. The calibration curve for oxytetracycline was obtained by plotting standard concentrations (0.15, 0.375, 0.75, 1.5, and 4.5 ng/mL) against relative absorbance (Figure-2) and the coefficient of determination was 0.9866. Out of the 22 samples, five (2.33%) were positive for oxytetracycline residues. The mean concentration of oxytetracycline residues in five positive pooled raw milk samples was estimated to be 201.00±41.25 ng/mL and residues ranged from 50.29 to 283.98 ng/mL using ELISA. The statistical analysis using one-way ANOVA showed no significant difference in the occurrence of the mean concentration of oxytetracycline residues between three blocks of Palakkad, Kerala (Table-2).

**Table-2:** Occurrence of oxytetracycline residues in pooled raw milk samples using ELISA.

Sl. no.	Blocks	Samples analyzed	Positive samples	Mean concentration±SE (ng/mL)	Range (ng/mL)	Samples above MRL
	
			No. (%)			No. (%)
1.	Alathur	9	3 (4)	200.49±75.25	50.29-283.98	2 (2.67)
2.	Chittoor	8	1 (1.43)	196.63	-	1 (1.43)
3.	Palakkad	5	1 (1.43)	206.9	-	1 (1.43)
Total		22	5	201.00±41.25	50.29-283.98	4

ELISA=Enzyme-linked immunosorbent assay, MRL=Maximum residue limits

**Figure-3:**
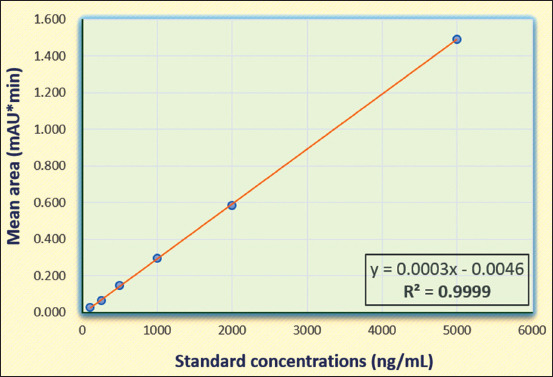
Standard calibration curve for oxytetracycline by high-performance liquid chromatography.

**Figure-4:**
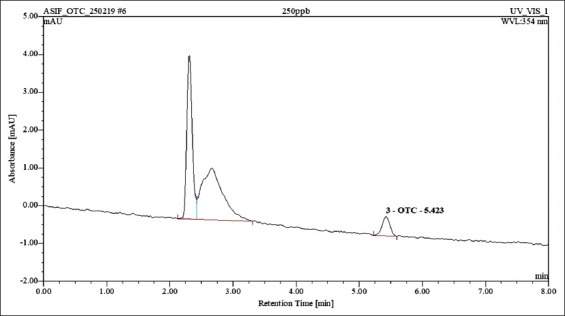
Chromatogram for 250 ng/mL oxytetracycline standard by high-performance liquid chromatography.

The standard calibration curve was obtained by plotting standard oxytetracycline concentrations of 50, 100, 250, 500, 1000, and 2000 ng/mL against average peak areas (Figures-3 and 4) and the coefficient of determination obtained was 0.9995. The intra-assay and inter-assay precision were found to be 4.10, 6.80, and 2.82% RSD and 7.61, 0.14, and 3.37% RSD for 200, 1000, and 2000 ng/mL fortified milk samples, respectively. The accuracy of the method for oxytetracycline residues varied from 88.65 to 94.75% recovery and the mean was 91.20% recovery. The LOD and LOQ obtained for the method were 82.65 and 250.45 ng/mL. The method validation parameters are listed in Table-3 and are in accordance with the European Commission Decision 2002/657/EC except for LOQ which is higher than maximum residue limits (MRL) of 100 ng/mL. The present standardized and validated HPLC method was applied to confirm oxytetracycline residues in pooled raw milk samples (Table-3).

**Table-3:** Validation parameters for HPLC.

Sl. no.	Parameter	Values for oxytetracycline
1.	Retention time (min)	5.4
2.	Linear regression equation	y=0.0003x−0.0046
3.	R^2^	0.9995
4.	Linear range (ng/mL)	50-2000
5.	LOD (ng/mL)	82.65
6.	LOQ (ng/mL)	250.45
7.	Intra-assay precision (RSD %) n=6 (ng/mL)	
	200	4.10
	1000	6.80
	2000	2.82
8.	Inter-assay precision (RSD %) n=6 (ng/mL)	
	200	7.61
	1000	0.14
	2000	3.37
9.	Accuracy (recovery %) n=6 (ng/mL)	
	200	90.19
	1000	88.65
	2000	94.75

HPLC=High-performance liquid chromatography, LOD=Limit of detection, LOQ=Limit of quantitation, RSD=Relative standard deviation

**Figure-5:**
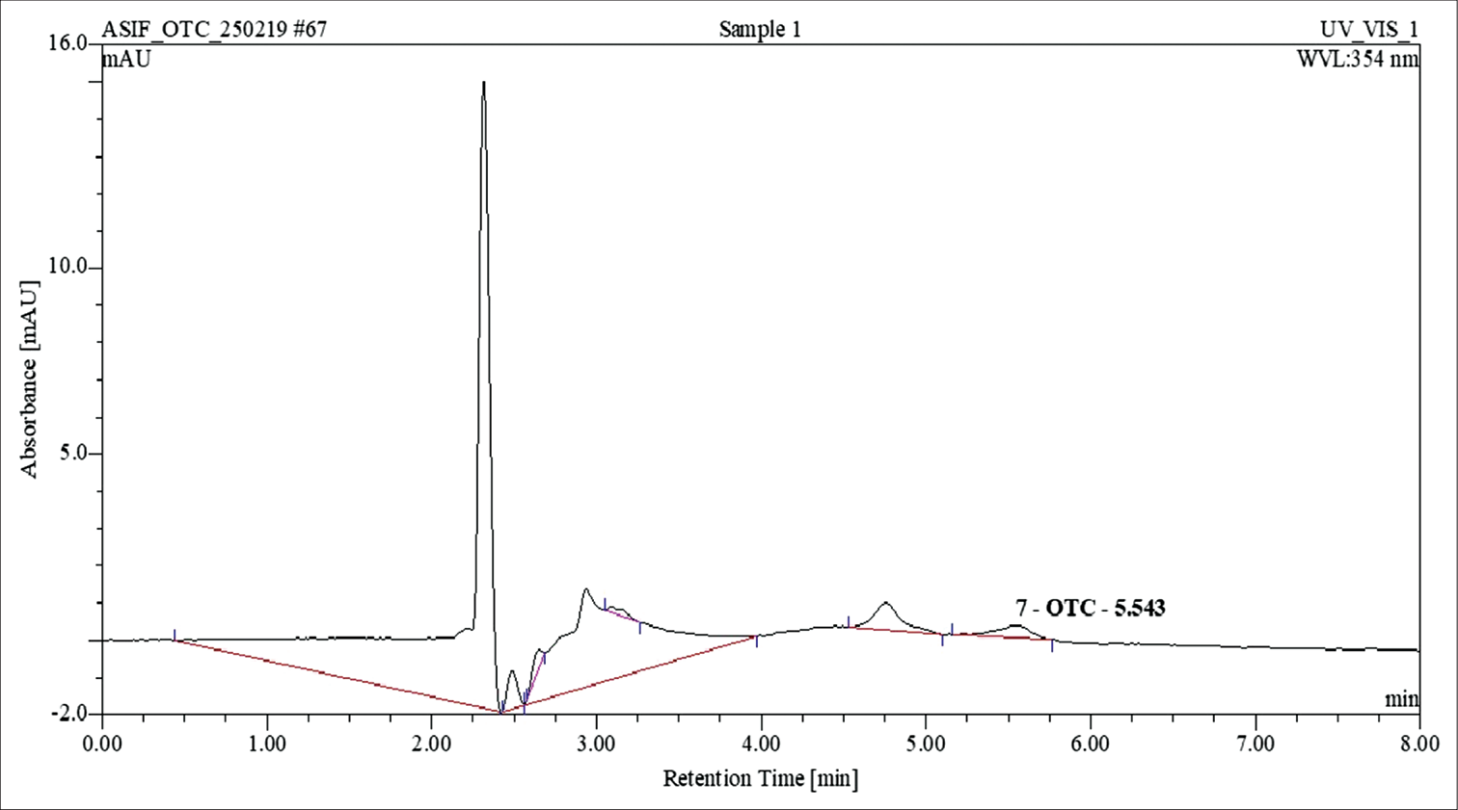
Chromatogram for oxytetracycline residues positive milk sample by high-performance liquid chromatography.

**Figure-6:**
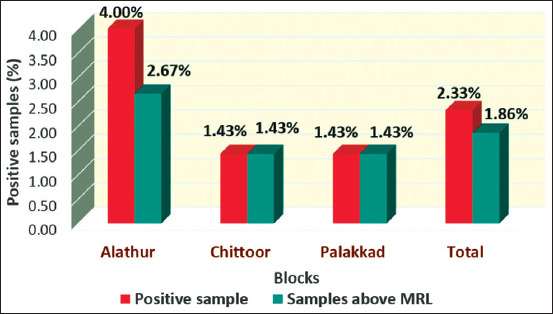
Occurrence of oxytetracycline residues in pooled raw milk samples of Palakkad.

All the five samples positive for oxytetracycline residues using ELISA were confirmed using HPLC and the occurrence of oxytetracycline residues in pooled raw milk samples of Palakkad, Kerala, was 2.33%. The mean concentration of oxytetracycline residues in five positive pooled raw milk samples was estimated to be 272.11±53.21 ng/mL and residues ranged from 91.86 to 366.45 ng/mL using HPLC (Figure-5). Out of the five positive pooled raw milk samples for oxytetracycline residues, four samples (1.86%) were above MRL established by the Codex Alimentarius Commission (CAC) of 100 ng/mL (Figure-6 and Table-4).

**Table-4:** Occurrence of oxytetracycline residues in pooled raw milk samples using HPLC.

Sl. no.	Blocks	Samples analyzed	Positive samples	Mean concentration±SE (ng/mL)	Range (ng/mL)	Samples above MRL
	
			No. (%)			No. (%)
1.	Alathur	9	3 (4)	256.71±83.91	91.86-366.45	2 (2.67)
2.	Chittoor	8	1 (1.43)	215.87	-	1 (1.43)
3.	Palakkad	5	1 (1.43)	374.57	-	1 (1.43)
Total		22	5	272.11±53.21	91.86-366.45	4

MRL=Maximum residue limits, HPLC=High-performance liquid chromatography

## Discussion

Microbiological assays are the screening methods employed for qualitative or semi-quantitative detection of antibiotic residues from milk samples [18]. They are cost-effective and are suitable for testing a large number of milk samples. We optimized the MIA with four standard cultures in four different media based on the work by Gaudin *et al*. [16]. The tested microorganisms, i.e., *B. cereus* showed more sensitivity for tetracyclines, *B. subtilis* for aminoglycosides and macrolides, *E. coli* for quinolones, and *G. stearothermophilus* for penicillins, cephalosporins, and sulphonamides [16,19]. We selected these four microorganisms to test the increase in sensitivity for the detection of a wide range of antimicrobial groups. After incubation, the presence of a zone of inhibition measuring 12 mm or more around the test disk (6 mm diameter) in at least one of the plates was considered as positive for antibiotic residues. The zone of inhibition reported for positive samples was 2 mm or more by Gaudin *et al*. [16] with 9 mm disks, while 22 mm by Nouws *et al*. [19] with 14 mm diameter punch holes.

Out of the 215 pooled raw milk samples screened, 22 samples (10.23%) produced 12 mm or more zone of inhibition in at least one plate. Arora and Chhabra [20] reported a 23.8% occurrence of antibiotic residues in unorganized dairy farms of Indore, Madhya Pradesh using *B. subtilis* on nutrient agar. A similar study conducted in Guwahati, Assam [21] reported a 23.3% occurrence of antibiotic residues in milk samples using *B. subtilis* on nutrient agar. A higher occurrence of antibiotic residues (49.75%) was reported in Sindh Province, Pakistan [22]. The widespread use of antimicrobials in livestock production system has led to the occurrence of antimicrobial residues in food of animal origins such as milk and meat [23,24]. Microbiological assays are an essential and efficient screening method for the surveillance and monitoring of antimicrobial residues in milk.

The detection and quantitation of oxytetracycline residues among positive pooled raw milk samples for antimicrobial residues were based on a competitive colorimetric ELISA. We standardized the ELISA by plotting a standard calibration curve with standard concentrations against relative absorbance. The detection limit of the assay was 1.5 ng/mL and the coefficient of determination (R^2^) obtained was 0.9866. The extent of cross-reactivity of the assay for the analytes oxytetracycline, chlortetracycline, minocycline, tetracycline, and doxycycline was 100%, 108%, 79%, 124%, and 62%, respectively. The quantitative biotin-avidin mediated competitive ELISA was developed by Jeon *et al*. [25] with LOD 48 ng/mL and R^2^ of 0.9862. A similar study conducted by Chen *et al*. [26] generated a monoclonal antibody against tetracyclines with detection ranging from 0.26 ng/mL to 2 ng/mL in milk.

The present study revealed to us that the occurrence of oxytetracycline residues was 2.33% (five samples) using the oxytetracycline ELISA in pooled raw milk samples of Palakkad district. The oxytetracycline residues ranged from 50.29 to 283.98 ng/mL and the mean concentration was estimated to be 201.00±41.25 ng/mL. Of the five positive samples, four were above MRL of 100 ng/mL established by the CAC/Food Safety and Standards Authority of India (FSSAI). Gaurav *et al*. [27] reported 16 to 134.5 ng/mL (13.5%) of tetracycline residues in milk samples using ELISA in Punjab. Kamberi and Sulaj [28] reported 5.8% of oxytetracycline residues in raw milk from Macedonia, of which 3.6% of samples were above MRL of 100 ng/mL. A higher occurrence of 86.4% of tetracycline residues was recorded in milk from Lebanon by Kabrite *et al*. [29]. The studies conducted for the detection of oxytetracycline residues in milk in Chhattisgarh [30], in Punjab [31], and in Haryana [32] were 278, 77, and 12.45 ng/mL, respectively. This presence of oxytetracycline residues confirms the widespread use of tetracyclines in bovines. The broad-spectrum activity, cost-effectiveness, and the easy availability of oxytetracycline could be the main reason for the wider use of this antimicrobial agent.

The liquid-liquid extraction and solid-phase extraction (SPE) are methods commonly used for the extraction of oxytetracycline residues from milk. The SPE consists of pre-packed cartridge columns that are conditioned and loaded with milk samples, then washed and eluted with appropriate solvents. The SPE methods employ long, laborious techniques, and columns are expensive. In the present study, we used 10% (w/v) trichloroacetic acid to coagulate milk proteins [33] and centrifugation was at a higher speed to separate solvent. The extraction procedure that we employed for oxytetracycline in this study involved a rapid, economical, and simple liquid-liquid extraction method.

The development of quantitative methods to assess antibiotic residues in milk is crucial to ensure milk safety and to establish surveillance systems. The tetracycline compounds have been detected using ultraviolet in milk [34], and the HPLC-DAD method was developed and validated in the present study using a reverse-phase C18 column. The HPLC method for the detection of oxytetracycline residues in milk consisted of mobile phase 0.03 *M* oxalic acid: acetonitrile: methanol in the ratio 70:15:15 delivered at 1 mL/min flow rate. The DAD was set at 354 nm for better quantification of residues. In a study conducted by Cinquina *et al*. [17], the mobile phase consisting of 0.01 *M* oxalic acid, acetonitrile, and methanol (60:25:15) was delivered at a flow rate of 0.6 mL/min. Abbasi *et al*. [35] also used a mixture of 0.05 *M* oxalic acid, methanol, and acetonitrile (70:10:20) as a mobile phase at a flow rate of 1 mL/min. Oxalic acid is used for the separation of tetracyclines due to its chelating property with metal ions.

Recovery study was conducted by fortification of blank milk samples at 200, 1000, and 2000 ng/mL oxytetracycline standard concentrations for evaluating LOD, LOQ, precision, and accuracy. This result is in accordance with Samanidou *et al*. [36] and Sreelekha [37], who observed 96.8 to 103.7% and 90.6% recovery for oxytetracycline residues in milk samples, respectively. Cinquina *et al*. [17], Furusawa [38], and Prado *et al*. [39] reported lower recoveries for oxytetracycline with 81.1, 80. and 82.5%. respectively. The precision and accuracy observed under the current study agree with the EU guidelines (<15-23% RSD and 80-120% recovery).

The occurrence of oxytetracycline residues was 2.33% using HPLC in pooled raw milk samples. All the five samples tested positive for oxytetracycline residues using ELISA were confirmed on analysis with HPLC. The oxytetracycline residues ranged from 91.86 to 366.45 ng/mL and the mean concentration in pooled raw milk samples was 272.11±53.21 ng/mL using HPLC. Out of five samples analyzed, four samples (1.86%) exceeded the MRL level of 100 ng/mL for oxytetracycline residues in milk specified by CAC/FSSAI. Kumar [30] reported the occurrence of oxytetracycline residues in 11% of the milk samples from Chhattisgarh, among which 7% of the samples were found to be above MRL with a mean concentration of 278 ng/mL. Nirala *et al*. [40] and Chauhan *et al*. [32] also have reported occurrence of oxytetracycline residues in 0.8 and 6% of milk samples in Bihar and Haryana, respectively. A study conducted on milk samples from Punjab [31] found oxytetracycline residues in 4.9, 4.3, and 3.7% from large, medium, and small dairy farms with a mean concentration of 94.8, 89.2, and 101 ng/mL oxytetracycline residues, respectively, using HPLC. Navratilova *et al*. [41] detected oxytetracycline residues in 50.6% of bulk milk samples in the Czech Republic, which is higher than the present study. Abbasi *et al*. [35] reported 10.5% of milk samples from Iran contained oxytetracycline residues. Kaya and Filazi [42] and Abebew *et al*. [43] reported 150.4 ng/mL and 125.25 ng/mL of oxytetracycline residues in milk samples from Turkey and Israel, respectively.

Typically, a withdrawal period of 96 h should be followed in dairy cows, which are treated with oxytetracycline. The occurrence of oxytetracycline residues in milk proves an insufficient observance of this withdrawal period in treated animals.

The present study confirmed the occurrence of oxytetracycline residues in 2.33% of pooled raw milk samples, of which 1.86% of the samples were above MRL in Palakkad, Kerala. This noncompliance of the withdrawal period and lack of awareness among farmers regarding the health effects and public health significance of antibiotic residues have led to the production of unsafe milk. The consumption of food with oxytetracycline residues above MRL and chronic exposure can cause lung congestion, liver injury, and damage to bones and teeth [44]. The occurrence of antimicrobial residues can be attributed to extra-label use and non-prudent usage of antimicrobials by para-veterinarians and farmers can be attributed to the occurrence of antibiotic residues.

## Conclusion

The present study revealed the occurrence of oxytetracycline residues in pooled raw milk samples in the Palakkad district of Kerala. The emergence of AMR and health effects of antimicrobial residues in milk is a threat to public health. There is a need for creating awareness among farmers, para-veterinarians, and pharmacists on misuse of antimicrobial agents leading to residues and implementing strict regulations on the sale of antimicrobial agents. There is also a need for establishing surveillance and monitoring systems to analyze real-time data with suitable laboratory infrastructure for consumer safety.

## Authors’ Contributions

MAH collected the samples and carried out the laboratory work. CL supervised the research work, reviewed the manuscript, and provided guidance for research work. KVM and JD contributed to the analysis and revised the manuscript. All authors read and approved the findings of the manuscript.
